# Following the Beat: Imaging the Valveless Pumping Function in the Early Embryonic Heart

**DOI:** 10.3390/jcdd9080267

**Published:** 2022-08-15

**Authors:** Shang Wang, Irina V. Larina

**Affiliations:** 1 Department of Biomedical Engineering, Stevens Institute of Technology, Hoboken, NJ 07030, USA; 2 Department of Integrative Physiology, Baylor College of Medicine, Houston, TX 77030, USA

**Keywords:** embryonic heart, valveless pumping, four-dimensional imaging, functional imaging, cardiodynamics, hemodynamics, confocal microscopy, bright-field microscopy, optical coherence tomography, light-sheet microscopy

## Abstract

In vertebrates, the coordinated beat of the early heart tube drives cardiogenesis and supports embryonic growth. How the heart pumps at this valveless stage marks a fascinating problem that is of vital significance for understanding cardiac development and defects. The developing heart achieves its function at the same time as continuous and dramatic morphological changes, which in turn modify its pumping dynamics. The beauty of this muti-time-scale process also highlights its complexity that requires interdisciplinary approaches to study. High-resolution optical imaging, particularly fast, four-dimensional (4D) imaging, plays a critical role in revealing the process of pumping, instructing numerical modeling, and enabling biomechanical analyses. In this review, we aim to connect the investigation of valveless pumping mechanisms with the recent advancements in embryonic cardiodynamic imaging, facilitating interactions between these two areas of study, in hopes of encouraging and motivating innovative work to further understand the early heartbeat.

## 1. Cardiac Pumping and Cardiovascular Morphogenesis

As the first organ to form and function during development, the embryonic heart plays critical roles through its pumping dynamics. Of particular interest is its role in morphogenesis, where biomechanical forces generated from the early heartbeat regulate the developing cardiovascular system [1,2,3,4,5]. In fact, with evidence showing that the cardiac contraction starts before the convective transport is needed [6], mechanical regulation of cardiovascular development is increasingly recognized as a significant function of early cardiodynamics.

**Figure 1:**
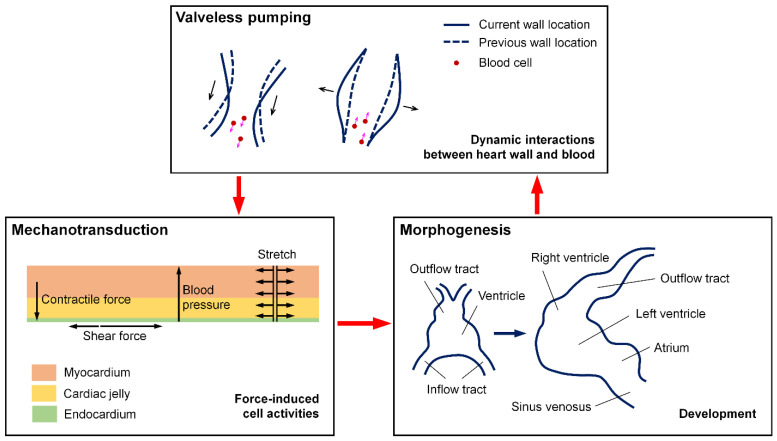
An illustration of the interplay between the cardiac pumping function and cardiovascular morphogenesis with mechanotransduction during early development.

While the early heartbeat produces several mechanical cues, the focus over the past two decades has mainly been on the flow-induced forces applied to the endothelial and endocardial cells [7,8,9,10]. Studies elucidated the intriguing role of the hemodynamic forces in regulating various aspects of cardiogenesis and angiogenesis through mechanotransduction, where multiple signaling pathways are activated upon force exposure. The level, pattern, and direction of the flow were all suggested as important factors in cardiovascular development, with different mechanosensing mechanisms involved [5]. For example, a decreased retrograde flow fraction (decreased oscillatory flow) leads to a reduction in *klf2a* expression in the atrioventricular canal through a mechanosensitive ion channel, which affects the valve formation [11,12]. The flow-induced forces are heterogeneous spatially and dynamic on different time scales; both intravascular and intracardiac blood flows present significant spatiotemporal variations as a result of the continuous and dramatic morphological changes in the early developing heart [13]. Figure 1 shows the interplay between the pumping function and the cardiovascular morphogenesis with the contraction/flow-induced forces and mechanotransduction during development. All these point to the need of understanding the cardiac pumping process, which could reveal the interconnection and regulation between the morphology and function of the heart during early development.

## 2. The Valveless Heart as a Pump: Insights Enabled by Imaging

Studies of valveless pumping dynamics were performed with avian, fish, and mouse models, largely enabled by the advancements in optical imaging. From early qualitative observations to recent quantitative assessments, the imaging resolution, speed, depth, and contrast have all been important factors pushing the limit of what information can be achieved from the beating embryonic heart. Behind the evolving knowledge of the valveless pumping mechanism is the development and application of new imaging modalities for probing cardiodynamics and hemodynamics.

The history of research into cardiac pumping at the early embryonic stage has been thoroughly reviewed by Männer et al. [14]. Direct observations of the contraction wave traveling throughout the heart tube in the chick embryo led to the characterization of the early tubular heart as a peristaltic pump [15,16]. Since the 21st century, new optical imaging capabilities have brought a novel understanding of pumping dynamics at the early tubular stage and also extended the assessment of valveless pumping beyond this stage, into the more complicated, looping heart.

**Line-scan confocal microscopy.** The revolutionary improvement of confocal microscopy from point scanning to line scanning enabled fast 2D dynamic (2D+time) imaging over 100 Hz [17]. This, along with 4D (3D+time) cardiodynamic reconstruction [18], has allowed unprecedented volumetric visualizations and quantifications of the heart wall movement and intracardiac blood flow in the zebrafish model [19]. Such analyses challenged the classical views of the tubular heart as a technical peristaltic pump by defining detailed wall–blood interactions, including the single active contraction site, the peak blood flow speed exceeding the speed of the contraction wave, a nonlinear frequency–flow relationship, and a negative pressure gradient giving rise to the maximal acceleration of flow [19]. As a result, a new suction pumping mechanism (impedance pump) of the tubular heart was proposed [19].

**Ultrafast bright-field imaging.** Relying on 2D bright-field imaging of zebrafish embryos, the use of an ultrafast camera has pushed the imaging speed to 1000–1500 frames per second while maintaining a micro-level pixel scale and a sufficiently large field of view [20]. The high speed and pixel resolution of the cardiodynamic video has made it possible to quantify a range of functional parameters through image processing methods and mechanical models [21,22,23]. These include the blood velocity, flow pattern, heart wall kinematics, luminal diameter, and intracardiac pressure field. Measurements of these parameters over developmental stages suggest a significant transition of the pumping mechanics from the wave-driven pumping at the early tubular stage to the displacement pumping with independent, full-chamber contractions at the late looping stage [21]. Analyses of the pressure gradient inside the heart and its relationship with the flow further describe the underlying pumping process of the looping heart and how retrograde flows are generated in the atrioventricular canal [22,23].

**Figure 2:**
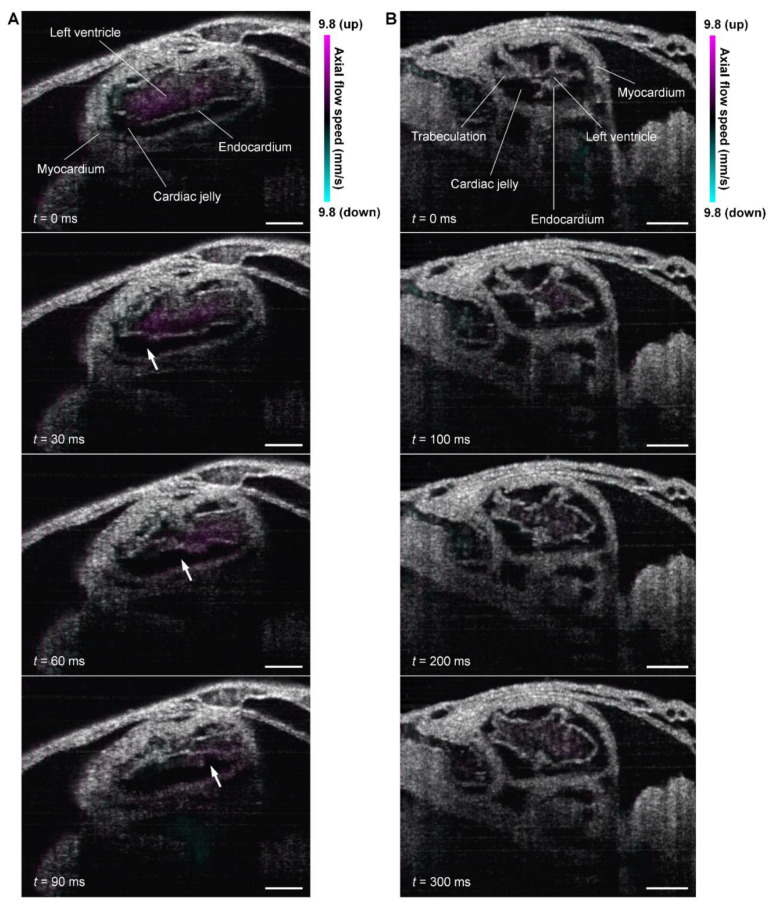
Four-dimensional OCT structural and Doppler imaging of the beating embryonic heart in the live mouse embryos at embryonic day 9.25 with cross-sectional visualizations. (**A**) Cardiac contraction wave in the left ventricle with the wave front (arrows) moving from left to right. (**B**) Left ventricular expansion with blood filling into the left ventricle. Scale bars are 100 µm.

**Figure 3:**
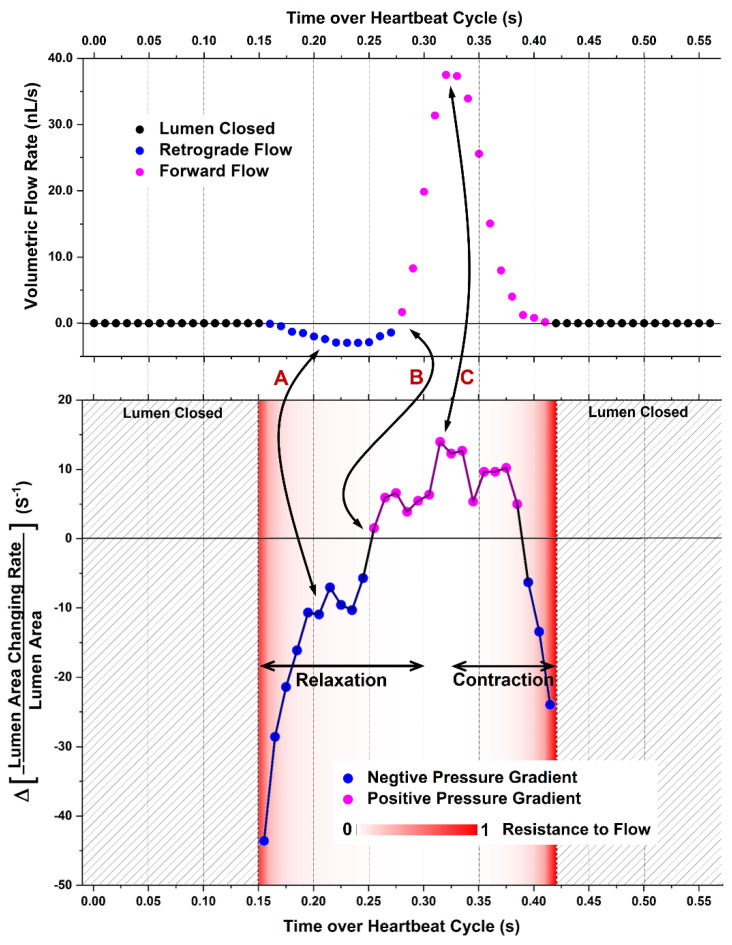
Plot of the temporal relation between the intracardiac pressure gradient, viscous resistance to flow, and volume flow rate for analyzing pumping dynamics in the early embryonic heart. This plot is from the right ventricle of an embryonic day 9.25 mouse embryo. Double arrows A–C indicate the corresponding changes in the flow and the pressure gradient. The pressure gradient is between two locations with a distance of ~60 µm, and the flow is measured around the middle of these two locations. Reproduced from [30].

**Optical coherence tomography (OCT).** With its fast micro-scale depth-resolving capability over a millimeter-level depth, OCT enabled 4D imaging of the embryonic heart in a range of model organisms [24,25,26,27]. Notably, the entire, live, beating embryonic heart in cultured mouse embryos can be imaged with a high spatiotemporal resolution using OCT [28] but is difficult to capture with most optical imaging modalities. Recently, as shown in Figure 2, 4D cardiodynamic and hemodynamic imaging of the looping mouse embryonic heart was performed to assess the pumping dynamics during mammalian cardiogenesis [29,30]. The pressure gradient induced by heart wall movements, the averaged viscous flow resistance, and the volumetric blood flow can be quantified at localized cardiac regions and were analyzed together to reveal the pumping process [30]. Figure 3 shows an example of such analyses. Specifically, the left and right ventricles of a looping heart present both pushing and suction processes at different phases of the heartbeat cycle [30], suggesting the coexistence of multiple pumping mechanics. Also, the flow in the outflow tract appears to not be driven by the local active contractions of the outflow tract [30], pointing to a spatial heterogeneity in how the valveless heart pumps blood.

Taking a further look at these three major imaging methods that have been utilized to study valveless pumping, the fast imaging speed has been the key in achieving targeted quantifications and information. Although the frequency of the embryonic heartbeat at this early stage is in the range of 1–3 Hz, a sampling rate that is ~50 times of the heartbeat frequency is necessary to resolve the detailed dynamics of cardiac activities. For example, high-speed confocal microscopy captured the bi-directional contraction waves and their reflections at the inflow and outflow tracts in the zebrafish tubular heart [19], which were undetectable with traditional imaging. Also, the subtle difference in the temporal profiles of luminal areas over a ~60 µm distance from the mouse embryonic heart leads to distinct area changing rates, contributing to the intracardiac pressure gradient [30], which can hardly be revealed with the slower imaging speed of OCT. In addition to fast imaging, the ability to simultaneously image heart wall movement and blood flow is also essential for pumping analysis. While the heart wall can be directly visualized and segmented from the imaging modalities, measuring the blood flow largely relies on processing of the imaging data. This is shown through cell tracking for confocal microscopy [19], microparticle imaging velocimetry for bright-field microscopy [23], and Doppler imaging for OCT [29].

There has been clear evidence showing that the early tubular heart is not a technical peristaltic pump [19]; however, categorizing the valveless embryonic heart as a well-defined type of pump has yet to be successful and might even be infeasible [14]. With the continuous and significant structural and morphological changes in the early developing heart, it is plausible that different pumping mechanisms function in different locations of the heart to drive blood circulations, especially during the looping process. Considering these, it is our perspective that, instead of looking to attribute the valveless heart to a defined pump, studies can focus on elucidating the detailed pumping process in a spatiotemporally resolved fashion. Regarding the temporal aspect, similar to the bright-field imaging study [21], the focus can be placed on the change or evolvement of pumping dynamics over cardiac development, linking the function with morphogenesis. Regarding the spatial aspect, the heterogeneity in how the blood is pumped arises as the heart loops [30], and assessment of specific sections of the heart becomes necessary. These contribute to painting the whole picture of valveless pumping, which would require further advancement of approaches. In the following three sections, we comment on selected aspects that could potentially make an impact in this endeavor.

## 3. Frontiers of Imaging with Light-Sheet Microscopy

The improvement in imaging speed from confocal microscopy has taken the dynamic assessment of the beating embryonic heart to a new level [31]. By achieving ultrafast imaging at a subcellular resolution over a larger depth, light-sheet microscopy holds strong promise for further advancing the analysis of valveless pumping dynamics in the zebrafish model [32]. This is shown by its advantages in both the imaging scale and imaging speed in comparison to confocal microscopy, while also maintaining the robust fluorescent contrast.

Regarding the scale, a higher imaging depth allows light-sheet microscopy to probe 4D structures and protein expressions of the whole heart at a later stage of zebrafish development [33], thus covering an extended range of the developmental period for analysis. Notably, 4D light-sheet imaging of cardiac morphogenesis was used to elucidate how flow-induced shear stress modulates the trabeculation through Notch signaling [34]. Furthermore, through 4D light-sheet assessment of the cardiac mechanics and valvular morphology, the relative contributions of the contractile force and hemodynamic shear force to the outflow tract valvulogenesis were revealed with corresponding mechanotransduction pathways [35]. Such studies laid the foundation for biomechanical analysis of the developing valveless heart, which can be directly applied to study the pumping process at a high resolution and throughout the entire pre-valve cardiogenesis.

With respect to the speed, development of advanced light-sheet microscopy enabled direct, volumetric imaging of the beating embryonic heart [36,37]. Focusing on tracking individual blood cells and imaging arrhythmic hearts, where post-acquisition synchronization was not applicable, Micholeit et al. employed a galvanometric mirror for rapid scanning of the light sheet and an electrically tunable lens for fast refocusing along the detection axis, which led to a 60 Hz volume rate of data acquisition, directly capturing the cardiodynamics and blood flow [36]. As a further advancement in speed, Voleti et al. recently established an oblique light-sheet approach for single-objective excitation and imaging, demonstrating ultrafast, direct, volumetric imaging of a beating heart at 100 Hz, while also maintaining submicron-to-micron spatial sampling [37]. Moreover, the system can achieve a 321-volumes-per-second direct imaging rate, which allows for tracking individual blood cells in the fast-flow region, revealing various behaviors at the single-cell level [37]. Such powerful ultra-high-speed volumetric imaging capabilities set the stage to bring significantly improved efficiency in analyzing cardiac pumping.

## 4. Modeling and Its Integration with Imaging

Computational mechanical modeling has been an important tool to investigate valveless pumping [38,39,40]. Numerical methods with a simulated embryonic heart allow for convenient changes in parameters, which, in most cases, can be hard to achieve in the imaging/manipulation experiment. Building a heart computationally requires data on the morphology, structure, dynamics, and material properties. Thus, high-resolution imaging has naturally been used to guide the modeling for numerical analysis, and this has provided important insights into the pumping behavior of early cardiogenesis. For example, incorporating two-way fluid–structure interactions, Kozlovsky et al. developed a heart-like model simulating the chick tubular embryonic heart and discovered that the early chick heart functions as a complex peristaltic pump with a duty cycle, termed as a biological pump [41]. To investigate the mechanics of tubular heart pumping, Sharifi et al. modeled the myocardium as a neo-Hookean material and identified the effect of wall stiffness on the cardiac function, concluding with a myocardium elasticity on the order of 10 kPa for the optimal pumping output [42].

Current integrations between modeling and imaging have largely been one-way: the imaging-instructed modeling analysis. More explorations in the other direction, where insights from modeling studies are utilized to assist the direct experimental/imaging assessment, can further improve the existing approaches for more advanced pumping investigations. From the two noted studies above, the detailed, temporal profiles of cardiodynamics and hemodynamics from specific conditions of numerical models [41] can be used to guide experimental analysis, and the biomechanical characterization of pumping in modeling [42] could contribute to defining the extent of induced mechanical changes for experimentally investigating the role of stiffness in valveless pumping. In addition to numerical models, greater involvement of mechanical models can extend the existing measurements to the critical cardiac parameters that are not directly available from imaging. Specifically, quantifications of the intracardiac pressure field and pressure gradient were enabled by using established models with proper assumptions, which led to a new understanding of the valveless pumping process that would otherwise be hard to achieve [22,23,30]. Further efforts to integrate modeling and imaging in such ways are of critical value in elucidating key aspects of early cardiac function.

## 5. Emerging Concepts and Analyses

**Cardiac jelly in pumping.** In the early embryonic heart, cardiac jelly is a layer of extracellular matrix between the myocardium and endocardium, supporting the overall cardiac morphology [43]. This jelly-like substance has long been researched for its roles in early cardiogenesis [44], while studies have largely focused on the structural and molecular aspects [45]. The function of the cardiac jelly in supporting the pumping process, specifically for reaching both the end-systolic luminal occlusion and a diastolic luminal opening, was first proposed by Barry [46]. However, there has largely been a lack of experimental or numerical analyses to elucidate how the cardiac jelly contributes to the mechanics of pumping. Recently, Männer and Yelbuz speculated that the noncircular cross-sectional shape of the endocardial lumen resulted from the nonuniform distribution of the cardiac jelly functions to increase the pumping efficiency of the valveless heart as it developed [47]. This points to an interesting direction for imaging-based investigations, especially with animal models that have cardiac jelly deficiency [48,49,50].

**Mechanical signaling for contraction.** Waves of cardiac contractions are essential for valveless pumping, but the long-believed electrical signaling among cardiomyocytes was recently challenged by a novel concept of mechanical signaling [51]. In this new framework, high strains trigger intracellular release of Ca^2+^ in local cardiomyocytes for contraction, which activates the neighboring cardiomyocytes through strains, thus propagating the contraction [51]. This explains the stiffness-dependent wave speed of the embryonic heart contraction and is supported by the experimental evidence that embryonic hearts continue to beat with disrupted electrical conduction [51]. While the definite signaling mechanisms and underlying molecular mechanisms remain to be determined, this direct mechanical coupling among cardiomyocytes to form contraction waves should be taken into account for future analysis of the pumping dynamics. In particular, this could affect the interpretations of the heart wall dynamics or mechanics when assessing their relationship with the blood flow.

**Causal analysis and active/passive wall dynamics.** Assessing interactions between heart wall movements and blood flow is essential to describe and understand the valveless pumping process. Temporal profiles of parameters measuring the wall and blood dynamics are frequently quantified and analyzed in experimental studies [19,22,30]. For example, the pressure–flow relationship in the tubular zebrafish heart was assessed to reveal a suction process of pumping [19]. However, the analysis of the causal relation between the temporal data was largely based on the observation of the plots and time (or phase) delays. Recently, the Granger causality test was employed for statistical evaluations of the causal relations between pressure and flow at different cardiac regions of mouse embryos, and this helped to statistically define the difference in pumping between the ventricles and the outflow tract [30]. The Granger causality test is a prediction-based method for time-series data and has found increasing applications in neuro studies [52]. Utilizing such a statistical testing method alongside the biomechanical interpretation of the heartbeat is expected to provide a more robust pumping assessment. Moreover, the active or passive dynamics of the heart wall can potentially be evaluated statistically using this approach, which will bring improved efficiency in characterizing valveless pumping, as it is important to identify active/passive wall motions [8,19].

**In vitro models of the embryonic heart.** Advancements in tissue engineering techniques have enabled the creation of many types of artificial organs, including heart organoids for modeling cardiac development [53]. Recently, synthetic embryos created from mouse stem cells demonstrated a successful development to embryonic day 8.5 and a beating heart [54]. Such in vitro engineering work provides new and interesting opportunities to study the pumping function of the early heart in a more controlled way that allows for easy manipulations. Furthermore, insights from the pumping studies, including the detailed mechanisms, wall dynamics, and flow mechanics, can contribute to the engineering of an artificial embryonic heart or pumping structures. Notably, a pump-bot [55] inspired by the suction pumping mechanism of the tubular heart [19] was developed for microfluidic applications. This type of in vitro model could potentially be a valuable tool to further validate our understanding of valveless pumping.

**Multi-contrast and multimodality imaging.** As discussed in Section 2, the key factors in studying valveless pumping are the imaging speed and the ability to measure heart wall movement and flow dynamics. While these can be achieved with one imaging modality (e.g., OCT) or one imaging contrast (e.g., fluorescence), obtaining additional information is necessary to further investigate the biomechanics of the early embryonic heart. One such type of information is the elasticity of the heart, which largely defines the contractile outcome, affecting the intracardiac pressure gradient. The current understanding of the mechanical behavior in valveless pumping remains highly limited, being based primarily on computational analyses [42]. Microindentation and pipette aspiration were used to measure the stiffness of embryonic heart tissue [56,57,58]; however, applying such techniques to the live, beating heart is extremely challenging. The high-resolution biomechanical imaging field has recently witnessed the emerging of two optical techniques: optical coherence elastography (OCE) and Brillouin microscopy [59]. OCE relies on the phase of the OCT signal to resolve micro- or nano-scale displacement in response to mechanical loads [60] and thus can be integrated with 4D OCT imaging of embryonic cardiodynamics and hemodynamics, achieving multi-contrast imaging to extend the dimension of pumping analysis. Using a different mechanical imaging principle, Brillouin microscopy detects Brillouin-scattered light caused by the traveling microscopic acoustic wave whose properties depend on the material viscoelasticity [61]. Brillouin microscopy has been applied to the embryonic tissues in the zebrafish and mouse models [62,63,64]. With further improvements in the imaging speed, the combination of Brillouin microscopy with fluorescence-based confocal or light-sheet microscopy as multimodality imaging is a powerful method to assess both the dynamics and mechanics of early valveless cardiac pumping.

**Manipulation of pumping and optogenetics.** Manipulations of the heartbeat are a critical component in studying embryonic heart function. The early-stage, developing heart is tiny in size and extremely delicate, and thus, manipulating the heart while maintaining its live, beating state inside the embryo has been challenging. Direct and indirect manipulations have been developed and successfully utilized to investigate early cardiac functions. As summarized in Table 1, these include outflow tract banding in chick embryos that changes the wall motion and hemodynamics [65,66,67], infrared-laser optical pacing of embryonic quail hearts that controls the heart rate and increases retrograde flows [68,69], centrifugation of zebrafish embryos that introduces time-dependent alterations in the heart morphology and functions with either an increase or a decrease in retrograde flows [22,70], use of lidocaine in zebrafish embryo culture that alters the heartbeat pattern to a reduced heart rate for a reduction in retrograde flows [11], and adjusting the environmental temperature of zebrafish embryos to change the heart rate [19]. Such approaches can facilitate in-depth experimental studies of valveless pumping which otherwise require numerical simulations and modeling. Recently, optogenetic pacing of the embryonic heart has been achieved, enabling both increases and decreases in the heartbeat rate as well as cardiac arrest [71,72,73]. Particularly for the mouse model, optogenetic pacing was shown to induce hemodynamic changes in the heart [74], which, together with the possibility of highly-localized pacing or disruption, suggests a potentially useful integration with pumping analysis to further assess the pumping dynamics during mammalian cardiogenesis.

## Author Contributions

Conceptualization, S.W. and I.V.L.; writing—original draft preparation, S.W.; writing—review and editing, I.V.L.; All authors have read and agreed to the published version of the manuscript.

## Figures and Tables

**Table 1:** Methods for manipulating the valveless pumping function in the embryonic heart.

Manipulation Method	Animal Model	Location to Apply	Effect on Cardiac Pumping
Banding	Chick	Outflow tract of heart	Wall motion pattern [66]
			Hemodynamics [65]
Infrared pacing	Quail	Inflow of heart	Heartbeat rate [68]
			Retrograde flow [69]
Centrifugation	Zebrafish	Whole embryo	Cardiac preload [70]
			Retrograde flow [22]
Lidocaine in culture	Zebrafish	Whole embryo	Heartbeat rate [11] Heartbeat pattern [11]
			Retrograde flow [11]
Temperature of culture	Zebrafish	Whole embryo	Heartbeat rate [19]
			Retrograde flow [11]
Optogenetics	Drosophila	Whole heart	Heartbeat rate [71,72]
			Cardiac arrest [72]
Optogenetics	Zebrafish	Selected locations of heart	Heartbeat rate [73]
			Heartbeat pattern [73]
			Cardiac arrest [73]
Optogenetics	Mouse	Selected locations of heart	Heartbeat rate [74]
			Blood flow [74]

## Data Availability

Not applicable.
